# Antioxidant Effects of Bone Marrow Mesenchymal Stem Cell against Carbon Tetrachloride-Induced Oxidative Damage in Rat Livers

**Published:** 2014-11-01

**Authors:** M. Ayatollahi, Z. Hesami, A. Jamshidzadeh, B. Gramizadeh

**Affiliations:** 1 Transplant Research Center, Shiraz University of Medical Sciences, Shiraz, Iran; 2 Shiraz Institutes for Regenerative Medicine, Shiraz University of Medical Sciences, Shiraz, Iran; 3 Department of Biology, Science and Research Branch, Islamic Azad University, Fars, Iran; 4 *Department of Pharmacology and Toxicology, Pharmaceutical Sciences Research Center, Shiraz University of Medical Sciences, Shiraz, Iran*

**Keywords:** Liver failure, Mesenchymal stem cells, Transplantation, Carbon tetrachloride

## Abstract

Background: Liver fibrosis results from excessive accumulation of extracellular matrix, which affects liver function over time and leads to its failure. In the past, liver transplant was thought to be the only treatment for end-stage liver disease, but due to the shortage of proper donors other medical treatments have been taken into consideration.

Objective: To evaluate the therapeutic effects of bone marrow derived mesenchymal stem cells (BM-MSC) in CCl_4_ damaged rats.

Methods: Liver damage in adult male Wistar rats was induced with carbon tetrachloride (CCl_4_). The rats were divided into normal control group, receiving CCl_4_, and those receiving CCl_4_ + marrow derived-MSC. Human BM-MSC was isolated, cultured, and characterized. The rats were injected with xenograft MSCs into the hepatic lobes of the liver. In the eighth week, blood samples were taken from all groups. Histological examination and biochemical analyses were used to compare the morphological and functional liver regeneration among different groups. Measurement of lipid peroxidation and glutathione transferase activity was also performed.

Results: Histopathology and biochemical analyses indicated that local injection of human BM-MSCs was effective in treating liver failure in the rat model. Furthermore, oxidative stress was attenuated by increased level of GSH content after MSC transplantation.

Conclusion: Evidence of this animal model approach showed that bone marrow-derived MSCs promote an antioxidant response and support the potential of using MSCs transplantation as an effective treatment modality for liver disease.

## INTRODUCTION

Liver transplantation is the most important treatment for patients suffering from liver failure, but this method has limitations due to the shortage of appropriate donors. Clinical and laboratory studies have considered alternative therapies for these patients. Recently, mesenchymal stem cell (MSC) has been investigated with the prospect of treatment of acute and chronic liver diseases. Some studies provide clinical and experimental evidence suggesting that MSC transplantation can restore the liver function in acute and chronic damages [1,2]. Other studies have concentrated on hematopoietic cells and stem cells derived from the bone marrow (BM-MSC), which have a great ability for division and implantation in the liver of transplant recipient [3].

In the bone marrow, there are main populations of stem cells including hematopoietic stem cells, MSCs and multipotent adult progenitor cells [4]. A number of studies have proven that under appropriate environmental conditions, cells derived from the bone marrow can differentiate into hepatocytes both *in vivo* [5,6] and *in vitro* [7]. We have previously found that BM-MSCs can participate in differentiation of hepatocytes. *In vitro* differentiated MSCs using IGF-I are able to display advanced liver metabolic functions supporting the possibility to use them as potential alternatives to primary hepatocytes [8].

Administration of MSC can decrease the injury in the liver, lungs and heart by reducing inflammation, collagen deposition and rearrangement [9]. Sakaida, *et al* [10], and Fang, *et al* [9], reported that bone marrow cells or MSCs can reduce hepatic fibrosis caused by carbon tetrachloride (CCl_4_) in mice. However, the reversal of fibrosis by MSC is not completely known so far.

Stem cells have the ability not only to implant in the target tissues, but also to secrete many factors that could change or improve the function of the damaged tissue [11]. Given that after transplantation of the damaged tissues or organs, the stem cells are influenced by several factors such as inflammatory cytokines [12,13], and migration to the target organs after a successful transplantation; the cells can activate processes that lead to reconstruction of the damaged cells and tissues [14,15].

CCl_4_ is a more efficient hepatotoxic substance, the toxicity of which is based on the change in its bio-structure into two free radicals and plays an important role in hepatotoxicity, tissue damage and cell death [16]. Oxidative stress contributes to the pathogenesis of various diseases. Tightly regulated defense systems such as glutathione production have evolved to combat these stresses. 

There are many controversies on the main mechanisms through which cell-based therapy affects liver tissue repair. The possibility of using human MSCs to repair liver damage has not yet been evaluated. In this study, we investigated the therapeutic use of MSC transplantation on CCl_4_-induced liver failure in rats.

## MATERIALS AND METHODS

Chemicals

CCl_4_; sodium dodecyl sulfate; ethylene diamine tetra-acetic acid (EDTA); 5, 5’-dithiobis-(2-nitrobenzoic acid) (DTNB); tris, thiobarbituric acid (TBA); and trichloroacetic acid were purchased from Sigma Chemical Company, Germany. All other chemicals were of highest quality available in the market.

Isolation of human MSCs (hMSCs)

Human MSCs (hMSCs) were isolated using a method previously described [17]. Cells from human bone marrow (BM) were taken from several people from the posterior iliac crest bone by aspiration in Namazi Hospital. Then, they were mixed with complete medium (ratio of 1:1) and mononuclear cells were gently placed in a separate Falcon using density gradient method (Percoll, 1.073 g/mL). The mononuclear cells were then isolated from the BM by centrifuging at 2000 rpm for 20 min at 21 °C and were cultured in complete medium (Dulbecco’s modified Eagle’s medium [DMEM, Gibco/BRL]) with fetal bovine serum (10%). The cells were then incubated at 37 °C in a medium containing 5% humidity and CO_2_ for 12–14 days; during this period, they began to form colonies. Once the colonies developed (filling 80%–90%), the media were washed twice in PBS and the cells were trypsinized with 25% trypsin in 1 mM EDTA (Gibco/BRL) for five minutes at 37 °C. After centrifugation, the cells were suspended in supplemented medium with serum and incubated in a 25-cm^2^ flask. Culture media were prepared for the first passage [18]. MSCs were identified in the culture medium by sticking and spindle deformation [19].

At each passage, the cells were counted and analyzed for viability by trypan blue staining analysis. Flow cytometric analysis and functional ability of differentiation into osteocyte and adipocyte was achieved in response to specific culture conditions. Each experiment described here was replicated thrice.

Preparation of animals and experimental groups

Twenty-one adult male Wistar rats weighing 250–300 g were purchased from Razi Institute for Serum and Vaccine and categorized into three groups (7 rats in each group). Group 1 rats (negative control) did not receive any CCl_4_; they only received olive oil intraperitoneally twice per week for eight weeks. Group 2 rats (positive control) received CCl_4 _diluted 1:1 in olive oil intraperitoneally (1 mL/kg) twice per week for eight weeks. Group 3 rats, in addition to receiving CCl_4_ intraperitoneally (1 mL/kg), received the prepared cells (1×10^6^ cells in 1 mL PBS) by insulin syringe into several lobes of the liver in a completely sterile environment in the fourth week after CCl_4_ injection. After the eighth week of CCl_4_ injection, the rats were anesthetized using thiopental (50 mL/kg). Their blood was collected for providing serum and their liver was dried after being washed with normal saline. Segments of their dried liver were collected for assessment of the oxidative stress and the rest was fixed in formalin (10%). Histological sections (5 μm) of the liver-lobes were obtained, stained with hematoxylin, eosin and Masson’s trichrome stain, and transported to the lab. 

Histopathological studies

A segment of the liver was excised from the animals and fixed in 10% formalin for at least 24 hours. Then, the paraffin sections were prepared and cut into 5-µm sections by a rotary microtome. The sections were stained with hematoxylin-eosin and studied for histopathological changes, *ie*, necrosis, fatty changes, ballooning degeneration, and inflammation. Histological damages were scored—0: “absent;” +: “mild;” ++: “moderate;” and +++: “severe.”

Measurement of serum alanine aminotransferase (ALT), aspartate aminotransferase (AST) and albumin 

Biocon standard kits and DAX-48 autoanalyzer were used to measure serum ALT, AST, and albumin, according to Wilkinson, *et al*, and Bessay, *et al*’s method [20,21].

Determination of lipid peroxidation

The extent of lipid peroxidation was assessed by measuring the amount of thiobarbituric acid-reactive substances (TBARs). In brief, 500 mg of the liver tissue was gently minced in 4.5 mL of 0.25 M sucrose. The minced tissue was gently homogenized and then centrifuged at 2000 rpm for 30 min. Afterwards, 0.1 mL of the supernatant was treated with a buffer containing 0.75 mL of thiobarbituric acid (0.8%, w/v), 0.75 mL of 20% acetic acid (pH 3.5) and 0.1 mL of sodium dodecylsulfate (8.1%, w/v). The solution was mixed up with 2 mL of distilled water and heated in a boiling water bath for 60 min. The absorbance was measured at 532 nm by a Beckman DU-7 spectrophotometer [22].

GSH determination

Glutathione reductase 5, 50-dithiobis-2 nitrobenzoic acid (DTNB) recycling procedure [23] was used to determine the reduced glutathione. In brief, 100 mg of the liver tissue was homogenized in a buffer containing EDTA (0.2 M) to obtain 4% (w/v) whole homogenate. Then, 1.5 mL of the suspension was taken and mixed with a buffer containing 2.5 mL distilled water and 0.5 mL of 50% TCA. The mixture was then centrifuged at 3000 rpm for 15 min, and 1 mL of the supernatant mixed with 1 mL of tris buffer (0.4 M, pH 8.9) and 0.1 mL of DTNB (0.01 M). The absorbance was measured after 5 min at 412 nm using a Beckman DU-7 spectrophotometer [24].

Statistical analysis

The data were analyzed by *Student’s t* test and one-way ANOVA, followed by Graphpad Prism 5. A p value <0.05 was considered statistically significant.

## RESULTS

**Figure 1:**
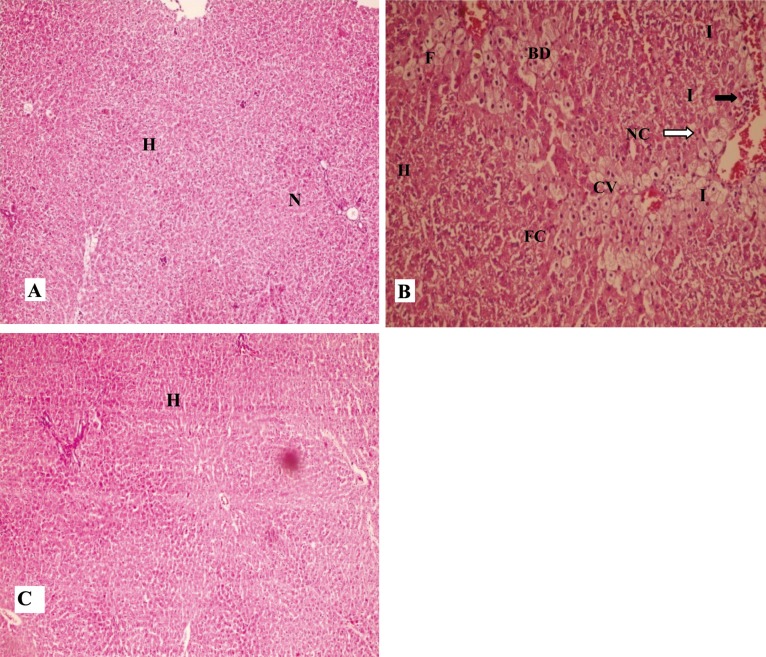
Effect of the bone marrow derived mesenchymal stem cell (BM-MSCs) on histopathological changes induced by CCl_4_ in rats. A: (H&E ×100) Liver section of normal rats showing normal hepatocytes with prominent nucleus, cytoplasm and central vein; B: (H&E ×250) Liver sections of CCl_4_-treated rats showing fatty chain, necrosis, infiltration of inflammatory cells; C: (H&E ×250) Liver sections of the rats treated with MSC showing well brought out central vein hepatocytes with well-preserved cytoplasm, normal hepatocytes with prominent nucleus. (H: hepatocyte, CV: central vein, N: nucleus, F: foamy macrophage cells, FC: fatty chain, NC: necrosis, I: infiltration of inflammatory cells, BD: ballooning degeneration).A: Control (0.5 mL/kg olive oil ip); B: CCl_4_ (0.5 mL/kg ip); C: MSC (106 cell in 1 mL PBS infusion in liver).

Histological studies of the liver sections from control rats showed normal architecture, characterized by polyhedral shaped hepatocytes with small uniform nuclei. Hepatocytes were arranged in well-organized hepatic cords and separated by narrow blood sinusoids. Hepatic sections, followed by CCl_4_ treatment, showed typical CCl_4_-induced hepatic injury (Fig 1B). The sections revealed extremely vacuolated hepatocytes (apoptotic necrotic cells and a lot of foamy cells) adjacent to the central vein (arrows and arrowhead, Fig 1B), indicating chronic CCl_4_-induced hepatotoxicity (with +++ grade, Table 1), moderate inflammation (with + grade, Table 1), and fatty changes (with + grade, Table 1). Hepatocytes were significantly protected from CCl_4_-induced toxicity after MSC infusion (Fig 1C), which demonstrated a normal appearance.

**Table 1:** Effect of the bone marrow-derived mesenchymal stem cell (BM-MSCs) on histopathological liver damages induced by CCl_4_ in rats

Groups	Ballooning Degeneration	Fatty change	Hepatocyte necrosis	Inflammation	Fibrosis
Control	0	0	0	0	__
CCl_4_	++	+	+++	+	Many foamy macrophages and old necrosis
CCl_4 _+ MSC	0	0	0	0	__

0: absent +: mild ++: moderate +++: severe

Rats were injected with CCl_4_ (1 mL/kg, ip 1:1 in olive oil) twice per week for 8 weeks. Four weeks after the first injection MSC was infused to their livers locally. The injection of CCl_4 _was continued until the end of 8^th^ week. Values are mean±SD of 6 rats per group.

The section from liver in Figure 1 shows severe necrosis in the central vein of the rats treated with CCl_4_ compared to the control group (with +++ grade, Table 1); there was no necrosis in the CCl_4_ + MSC treated group (with 0 grade, Table 1). Liver lobules in the CCl_4_ + MSC treated group had a relatively normal appearance, compared to the CCl_4_ group with no fatty changes (with 0 grade, Table 1 and Fig 1C). The effects of MSCs on reducing necrosis (with + grade, Table 1) surrounding the central vein area (Fig 1C), and the absence of swollen hepatocytes were clearly shown in the H&E sections. 

**Figure 2:**
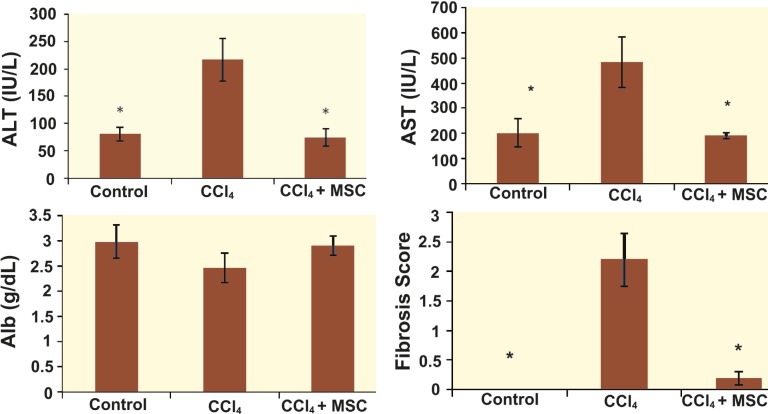
Effect of the bone marrow derived mesenchymal stem cell (BM-MSCs) on rat hepatic enzymes and albumin levels changed by CCl4. A: ALT                    B: AST                     C: Albumin                     D: Fibrosis scoreRats were injected CCl4 with doses (1 mL/kg ip 1:1 in olive oil) twice per week for 8 weeks. Four weeks after the first injection, MSC was infused to their livers locally and then injection of CCl4 was continued for 8 weeks. Values are mean±SD of 6 rats per group. *Significantly different from CCl4-treated group (p<0.05). **Significantly different from CCl4-treated group (p<0.01).^a^Fibrosis scores of different studied groups on Ishak scoring [33] graded from 1–6.

Histopathological examination of the liver revealed that MSC has anti-fibrosis effects by reducing the amount of collagen which is shown by Masson’s trichrome stain compared to the group receiving CCl_4_, which showed significant periportal fibrosis (Fig 2D).

The results showed slight increase in the serum albumin levels in the group receiving CCl_4_ + MSC compared to the group receiving CCl_4_ alone (Fig 2A). Other liver enzymes also showed significant changes. For instance, AST and ALT in the group treated with CCl_4_ + MSC were significantly (p<0.01) lower compared to the group receiving CCl_4_ alone (Figs 2B and 2C).

Lipid peroxidation in the liver showed a significant increase in the CCl_4_ group compared to the control group (p<0.01); in the CCl_4_ + MSC treated group, a significant reduction was observed in lipid peroxidation levels (p<0.01) (Table 2).

**Table 2:** Effect of the bone marrow-derived mesenchymal stem cell (BM-MSCs) on GSH and TBARs levels of the liver damaged by CCl4 in rats.

Groups	GSH (nMol/g liver)	TBARs (nMol/g liver)
Control	0.35454±0.035*	0.7±0.057**
CCl_4_ (0.5 mL /kg)	0.18727±0.016	4.96±0.84
CCl_4 _+ MSC	0.46±0.06*	0.59±0.012**

GSH: reduced glutathione; TBARs: thiobarbituric acid reactive substances

Values are mean±SD, (n=6).

* p<0.05 Mean difference, compared to CCl4-treated rats.

** p<0.001 Mean difference, compared to CCl4-treated rats.

Glutathione (GSH) was also assessed as an indicator of hepatic antioxidant enzymes. A significant increase was observed in GSH levels in CCl_4_ + MSC treated group compared with the CCl_4_ group (p<0.05). Moreover, a significant increase was observed in GSH levels in CCl_4_ + MSC treated group compared with the control group (p<0.05) (Table 2).

## DISCUSSION

Rats treated with CCl_4_ are usually used as an *in vivo* model for studies on liver damage. CCl_4_ usually produces free radicals that trigger a cascade of reactions leading to liver fibrosis. CCl_4_ is converted to free radicals by cytochrome P450 which exerts its effects on the liver through lipid peroxidation [25,26].

Oxidative stress is a pathogenic mechanism in the initiation and progression of liver damage involved in many liver disorders. Cell damage occurs when reactive oxygen species concentration increases in the liver. The use of antioxidants reduces the amount of these free radicals [27] .

Glutathione (l-γ-glutamyl-l-cysteinyl-glycine), which is present in all mammalian tissues, especially the liver, provides the reduction capacity for most reactions and plays a very important role in the detoxification of hydrogen peroxide, other peroxides and free radicals [28] .

In the present study, the hepatic content of GSH was found to be decreased significantly in the CCl_4_ intoxicated rats compared to the controls. Table 2 shows that MSC-based therapy significantly inhibited the CCl_4_-induced decrease of hepatic GSH content, and it was significantly increased in the MSC-treated group. The resulting hepatocellular toxicity has been demonstrated in numerous studies as reflected by increased liver enzymes. 

Because the liver is considered the main organ for biological degradation and subsequently detoxification of harmful substances, it contains important enzymes for their biological actions. When CCl_4_ is administered to rats, the activity of AST and ALT in plasma rises significantly with necrosis of and lipid accumulation in hepatocyte [29]. Both enzymes are indicators of liver injury. ALT is more sensitive to acute liver injury, whereas AST is more sensitive to chronic injury [30]. In this study, we investigated alteration of liver enzymes after transplantation of MSCs into the rat liver injured with CCl_4_. MSCs transplantation restored the increase of liver enzymes as well as the up regulation of albumin. 

Recovery of liver function after MSCs transplantation was also examined by histological changes. MSCs reversed the hepatic necrosis, fatty changes, and inflammation. However, the mechanisms by which MSCs repair liver damage remain unclear. HGF is one of the factors produced by the MSC and its beneficial effects include mitogens, morphogens, and anti-tumor activities [31]. Specifically, it was determined that HGF exerts beneficial effects on liver damage. MSC transplantation regenerates the reduction in hepatic protective genes and shows its effects by reducing LP, as determined by the amount of GSH in the liver. It was also determined that in ischemic/perfusion damage to liver, MSC transplantation leads to suppression of oxidative stress and reduction in the amount of apoptosis in rats [32].

We have shown that MSC transplantation may protect liver injury by altering the oxidative effect of CCl_4 _by increasing GSH content of the liver. In conclusion, we demonstrated the protective effect of MSCs, and suggest that bone marrow-derived MSCs could be a therapeutic approach for liver damage, particularly for those due to oxidative stress.
